# Phantom sensation and quality of life among patients with lower-limb amputations in the region of Juiz de Fora, Minas Gerais: a cross-sectional study

**DOI:** 10.1590/1980-57642021dn15-020016

**Published:** 2021

**Authors:** Víctor de Oliveira Costa, Fabrício Machado Teixeira, Thais Medeiros Lopes, Henrique Pinto Gomide, Patricia Cardoso Clemente, Demóstenes Moreira

**Affiliations:** 1 Faculdade de Ciências Médicas e da Saúde de Juiz de Fora - Juiz de Fora, MG, Brazil.; 2 Universidade Federal de Juiz de Fora - Juiz de Fora, MG, Brazil; 3 Department of Education, Universidade Federal de Viçosa - Viçosa, MG, Brazil; 4 Physiotherapy Department, Faculdade de Ciências Médicas e da Saúde de Juiz de fora - Juiz de Fora, MG, Brazil; 5 Orthopedics and Traumatology Department, Hospital Universitário Antônio Pedro, Universidade Federal Fluminense - Niterói, RJ, Brazil

**Keywords:** quality of life, phantom limb, lower extremity, amputation, qualidade de vida, membro fantasma, extremidade inferior, amputação

## Abstract

An amputation is an irreversible event that causes social, psychological, and functional consequences that reduces the quality of life of the amputee. Phantom pain generally is reported by 50 to 80% of amputees.

**Objective:**

To describe the pain and phantom sensation and quality of life among lower-limb amputees.

**Methods:**

This was a cross-sectional study carried out in the region of Juiz de Fora, state of Minas Gerais, Brazil. Inclusion criteria were being a patient in one of two hospitals in the region at the time of the interview and having at least one lower-limb amputation. A total of 20 amputees were included in the analysis. The interview questionnaire had items adapted from the Groningen Questionnaire Problems After Leg Amputation - describing the frequency and discomfort of phantom pain and sensation, causes and the level of the amputation, as well as the WHOQOL-BREF, for assessing quality of life.

**Results:**

Most participants were women (55%) and had a mean age of 55.6 years (SD=14.8). Femoral amputation was the most prevalent (65%), and diabetes (40%) was the main reason for amputation. 29% of amputees classified the phantom pain as moderate or severe, and 15% claimed daily frequency of this phenomenon. As for phantom pain, only 6% stated daily frequency. The mean quality of life was 4.1 (SD=1.1, five score means very satisfied), the physical domain of quality of life had the lowest mean (3.4, SD=0.7).

**Conclusions:**

Phantom sensation and pain were prevalent among lower-limb amputees who were, in general, less satisfied with their physical domain of quality of life.

## RESUMO.

A amputação é um procedimento irreversível que gera consequências sociais, psicológicas e funcionais. A dor do membro fantasma ocorre entre 50 e 80% dos amputados, apresentando implicações na qualidade de vida.

**Objetivo:**

Avaliar a sensação, dor fantasma e qualidade de vida entre pacientes amputados.

**Métodos:**

Trata-se de um estudo transversal retrospectivo realizado na região de Juiz de Fora, Minas Gerais, Brasil. Os critérios de inclusão foram possuir pelo menos uma amputação de membro inferior e ser paciente de um dos hospitais referência para região. Vinte participantes foram incluídos nas análises. O questionário da pesquisa consistiu em itens, adaptados do *Groningen Questionnaire Problems After Leg Amputation*, que descrevem frequência e incômodo da sensação fantasma, dor fantasma, causas e nível da amputação, e questões sobre os tratamentos além do questionário qualidade de vida WHOQOL-BREF.

**Resultados:**

A maioria dos participantes era do sexo feminino (55%) com idade média de 55,6 (DP=14,8) anos. A maior frequência de amputação foi transfemural (65%), e o principal motivo da amputação foi o diabetes mellitus (40%). Com relação ao incômodo da sensação fantasma, 29% a classificou como moderado ou severa, e 15% alegou frequência diária desse fenômeno. Quanto {a dor fantasma, apenas 6% afirmou frequência diária. A qualidade de vida média dos participantes foi de 4,1 (DP=1,1) em 5 (cinco significa muito satisfeito), de acordo com a primeira questão do WHOQOL-BREF; o domínio físico apresentou a menor média 3,4 (DP=0,7).

**Conclusão:**

A sensação e dor fantasmas foram prevalentes nos entrevistados que apresentaram menor satisfação com o domínio físico da qualidade de vida.

## INTRODUCTION

Amputation is an irreversible event that causes social, psychological, and functional consequences that reduce the quality of life of the amputee.^1^ According to an American National Survey, 95% of amputated patients reported experiencing one or more amputation-related pain; phantom pain was the most prevalent. However, epidemiological studies are still scarce in Brazil, especially in the region of Juiz de for a, state of Minas Gerais.^2^ Estimates of incidence in the worldwide literature vary from 2.8 to 43.9/105 inhabitants/year, and 85% of these amputations are performed on lower limbs (LL).^3^ ^,^ ^4^ According to data of the Brazilian Unified Health System (*Sistema Único de Saúde* - SUS), the main causes of amputation are external (33.1%), infectious and parasitic diseases (17.9%), diseases of the circulatory system (16.1%), and diabetes (13.6%).^3^

Regardless of the cause, amputation carries a dramatic change in people’s lives.^3^ ^,^ ^5^ Phantom limb pain is a complication that occurs in 50 to 80% of amputees, and phantom sensation occurs in 90% of patients within six months after surgery.^6^ ^,^ ^7^ They usually start in a few weeks following surgery, even though they can begin years later.^1^ ^,^ ^7^ Phantom limb sensation and pain can coexist with fantasy and pain sensations on the residual limb.^6^

Flor et al.^6^ define phantom pain as a neuropathic pain associated with central or peripheral neuronal lesions caused by the central sensitization from peripheral stimuli. At the cortical level, areas of the somatosensory and motor cortices are reorganized, with a reduction in receptive fields;^1^ ^,^ ^6^ psychological factors also contribute to the phenomenon.^6^ ^.^ ^8^ Moreover, personal and environmental factors play a role in determining the long-term functionality of an amputation.

In this context, it has already been demonstrated that psychosocial support helps to improve the quality of life of people living with amputations.^5^ Quality of life is one of the main goals of rehabilitation programs.^5^ ^,^ ^9^ In this study, the objective was to describe the phantom pain and phantom sensation and quality of life among lower-limb amputees. Specifically, the frequency and intensity of the phantom sensation are described and the association between the phantom pain and sensation and quality of life is evaluated. Furthermore, the main causes of amputation in the evaluated participants were evaluated as a secondary objective.

## METHODS

### Study design and setting

This was a cross-sectional descriptive study. Patients from two referral hospitals of the city of Juiz de Fora were interviewed, covering a population of approximately two million people in the Zona da Mata region of Minas Gerais, Brazil. One of these hospitals is the region’s reference center for prosthesis placement and rehabilitation of amputees. The interviews took place between August 2018 and February 2019, after the approval of the Institutional Review Board (Protocol #3.072.371-11/12/2018, Faculdade de Ciências Médicas e da Saúde - SUPREMA). After the participants’ consent, the authors’ proceeded to the interview.

### Participants

All patients who had been admitted to the two hospitals were invited to participate in the study. The inclusion criteria were:

- Being a patient in one of the hospitals.
- Having at least one lower-limb amputation, regardless of the cause of the amputation.
- Did not present a great discomfort or pain during the interview.

Participants with multiple amputations of both an upper and a lower limb and those unable to answer the questionnaire due to cognitive impairment or comprehension were excluded. Data from 20 participants (two interviews were prematurely finished) were reported at the end of the study.

### Instruments

World Health Organization Quality of Life-Bref (WHOQOL-BREF). The WHOQOL-BREF contains 26 items (the extended version has more than 100 items) that assess the quality of life.^10^ The first two items assess the quality of life and satisfaction with health, respectively; the following items are divided into four domains: physical, psychological, social relations, and the environment. WHOQOL-BREF is validated for multiple languages and health conditions - including phantom pain.^3^ ^,^ ^11^ The questionnaire showed good psychometric properties.^10^ ^,^ ^12^ ^,^ ^13^

Questionnaire for Lower-Limb Amputations. The 28-item questionnaire was created by the authors based on definitions and items from the Groningen Questionnaire Problems After Leg Amputation (QGPLA),^14^ which had been adapted from the Questionnaire Problems After Arm Amputation (GQPAA).^15^ Phantom limb pain was defined as any painful sensation in the missing part of the limb, whereas phantom limb sensation was defined as any unpainful sensation in the missing part of the limb. Stump pain was defined as any painful sensation in the stump.^15^ Items covered phantom sensation and pain, stump pain in the upper limbs, and the use of prostheses.^15^ This questionnaire is available upon request.

### Data analysis

Double data-entry was used to ensure that all items were correctly added to the database. Then, the exploratory data analysis was performed. Descriptive analysis was conducted using frequencies, percentages, central tendency measures (i.e., mean, median) and dispersion (i.e., interquartile range and standard deviation). WHOQOL-BREF scores were computed according to the algorithm of the World Health Organization (https://www.who.int/publications/i/item/WHOQOL-BREF). Statistical analysis was performed in R.^16^ Scripts and anonymous data are made available upon reasonable request.

## RESULTS

### Participants’ characteristics

Most participants were women (55%), with an average age of 55.6 years (SD=14.8). Concerning the amputation side, 12 participants had amputation on their right side, nine on their left side, and one participant had both lower limbs amputated. Regarding the level of the lower-limb amputation, most were transfemoral (65%), followed by transtibial (20%), knee-exarticulation (10%), and only one participant had a hip amputation (5%). The main reasons for amputations were diabetes (40%), trauma (30%), other vascular diseases (20%), and other reasons such as prosthesis rejection and tumor (20%). According to WHOQOL-BREF, most participants reported a good quality of life - mean value of 4.1 in a 5-point-scale (SD=0.8) and were satisfied with their health - mean value of 4.1 in a 5-point-scale (SD=1.1).

Among the domains (physical, psychological, social relations, and environment) evaluated by WHOQOL-BREF, the one with the lowest mean value was the physical domain, with a mean score of 3.4 (SD=0.7). Regarding the frequency of prosthesis use, 80% said they never used it and 20% use it daily, for eight hours or more. Table 1 describes the participants’ characteristics and quality of life domains.

**Table 1.** Demographics, level of amputation, and quality of life according to the World Health Organization Quality of Life-Bref questionnaire among lower-limb amputees (n=20).

	Mean (SD)	n
Sex [Women] (Fr., %)	11 (55%)	20
Age	55.6 (14.8%)	20
Laterality prior to amputation [Right] (Fr., %)	18 (94.7%)	19
Side of amputation (Fr., %)		20
Right side	11 (55.0%)	
Left side	8 (40.0%)	
Both	1 (5.0%)	
Level of amputation (Fr., %)		20
Transfemoral	13 (65%)	
Transtibial	4 (20%)	
Knee-exarticulation	2 (10%)	
Hip	1 (5%)	
Causes of amputation (Fr., %)		20
Diabetes	8 (40%)	
Trauma	6 (30%)	
Other vascular diseases	4 (20%)	
Other (prosthesis rejection, tumor)	4 (20%)	
Quality of life [WHOQOL-BREF]	4.1 (0.8%)	20
Satisfaction with health [WHOQOL-BREF]	4.1 (1.1%)	20
Quality of life domains [WHOQOL-BREF]		
Physical	3.4 (0.7%)	20
Psychological	3.9 (0.5%)	19
Social relationships	3.8 (0.8%)	20
Environment	3.6 (0.7%)	20

WHOQOL-BREF: World Health Organization Quality of Life - Bref version. Fr.: frequency; SD: standard deviation.

### Phantom sensation and pain

A total of 13 (70%) amputees reported phantom sensation: three felt it always or sometimes a the day; six (30%) described it a few times a week; five (25%) feel it sometimes a month or a year; and six (30%) reported they never had the phantom sensation. However, most amputees (59%) did not feel phantom sensation discomfort. Participants described their phantom sensation as itching (31.1%), movement (25%), electric sensation (25%), heat (6.25%), and other (12.5%).

**Figure 1.**
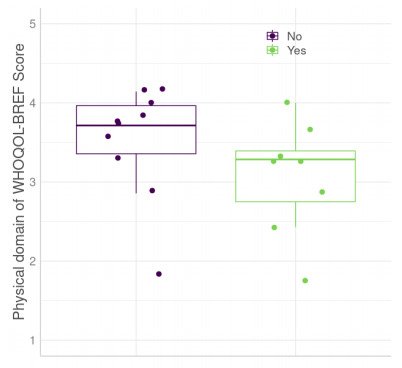
Comparison of World Health Organization Quality of Life-Bref physical domain scores between amputees with phantom pain and whithout panthom pain.

Seven participants (45%) said they suffered from phantom pain. Among the participants who suffered from phantom limb pain, five treated their phantom pain with physiotherapy, occupational therapy, and analgesics; three did not treat it. Amputees used different strategies to deal with phantom pain such as contracting the stump (25%), massaging the stump for some time (25%), rubbing the stump (25%), and pressing the painful region (25%). Figure 1 describes the differences in the physical domain scores of the WHOQOL-BREF between amputees who never felt phantom pain (median of 3.71, IQR=0.64) and those who felt it (median of 3.29, IQR=0.60).

WHOQOL-BREF: World Health Organization Quality of Life - Bref version.

Most of the amputees have never felt pain in the stump (55%); 25% felt it sometimes a month or a year; 15% felt it sometimes a week; and 5% daily felt it. A total of 70% of amputees did not receive treatment for stump pain. Phantom sensation and pain, as well as stump pain, are depicted in Table 2.

**Table 2.** Frequency and intensity of phantom sensation, phantom and stump pain among lower-limb amputees (n=20).

	Fr.* (%)	n
Frequency of phantom sensation		20
Always or sometimes a day	3 (15%)	
A few times a week	6 (30%)	
Sometimes a month or a year	5 (25%)	
Never	6 (30%)	
Intensity of discomfort caused by the phantom sensation		17
None	10 (59%)	
Rare	2 (12%)	
Moderate	4 (24%)	
Severe	1 (5%)	
Frequency of time suffering from phantom pain		18
Always or sometimes a day	1 (6%)	
A few times a week	4 (22%)	
Sometimes a month or a year	3 (17%)	
Never	10 (56%)	
Have you received treatment for phantom pain?		8
Yes (physiotherapy, occupational therapy, analgesics)	5 (63%)	
No	3 (37%)	
Frequency of stump pain		20
Always or sometimes a day	1 (5%)	
A few times a week	3 (15%)	
Sometimes a month or a year	5 (25%)	
Never	11 (55%)	

Fr.: frequency.

## DISCUSSION

The present study described the phantom sensation and pain among lower-limb amputees from the region of Juiz de Fora. Most amputees reported phantom sensation. The physical domain of quality of life had the lowest scores among the other domains, although, overall, the reported quality of life was perceived as good and amputees were satisfied with their health.

These findings are similar to Zidarov et al.^9^, who assessed lower limb amputees’ quality of life during rehabilitation. Although amputees reported a good quality of life, the physical domain had the lowest scores.^9^ As reported in Sinha et al.^5^, phantom pain negatively impacts quality of life in relation to other phantom events. The findings of these authors are similar to studies that included other amputations.^5^ Vand der Schans^8^ highlights that amputees who suffer from phantom pain had a worsened quality of life than amputees who do not suffer from it. This comparison was not possible in the present study due to the small number of participants.

Diabetes, trauma, and other vascular diseases were the main reasons for amputations. The present findings were similar to Kahle et al.^17^ They found the following causes of lower-limb amputations: 37% of peripheral vascular disease (PVD), 27% of trauma, 17% of diabetes, 12% of cancer, 6% of infection, and 2% of congenital diseases.^17^ Another study showed that the mean age of amputees was 70.6 years; the main cause of amputations was diabetes (44%).^18^ Spichler et al.^19^ described the main causes of lower-limb amputations in Rio de Janeiro, Brazil, in a retrospective study. According to the authors, 56.3% of amputations were caused by peripheral arterial disease and 43.7%, by diabetes mellitus. The major amputations of primary lower limbs were in the thigh (71.8%), 59.9% for peripheral arterial disease, and 40.1% for diabetes.^19^

Phantom sensation was prevalent among most amputees, although only a few of them reported discomfort or pain. The present findings were somewhat similar to those found in the literature. Esfandiari et al.^20^ observed the prevalence of phantom sensation (82%), phantom pain (63%), and stump pain (49%) among lower-limb amputees, while Yari et al.^21^ found a 73% prevalence of phantom pain among patients with hip disarticulation or hemipelvectomy. Almost two-thirds of the participants from Aldington et al.^22^ study reported phantom sensation; 41% classified their discomfort as moderate or severe. Participants with phantom pain treated it with physiotherapy, occupational therapy, and analgesics. They coped with other strategies such as massaging, rubbing, or pressing the stump.

This study has limitations. First, the results cannot be generalized by researchers. A small number of amputees was reached out due to the difficulty in recruiting participants with lower-limb amputations. To mitigate the low number, the collection period was extended by four months. Another limitation concerns the restriction of the type of amputation. In this study, exclusion criteria were defined as people with multiple amputations of both an upper and a lower limb, as phantom sensation and pain can be confounders. Another potential limitation is the lack of published adaptation and validation studies of the lower-limb questionnaire in Brazilian Portuguese, which is a potential source of bias. The QGPLA^14^ is a questionnaire that comprises a general description of phantom pain based on previous research; QGPLA^14^ is widely used in studies and does not contain any psychometric scale that requires further validation and assessment such as the WHOQOL-BREF.

 Therefore, according to the study findings, phantom sensation and pain were frequent among lower-limb amputees who sought treatment in the region of Juiz de Fora. Diabetes was the main reason for amputations, demonstrating its importance in the context of public health. Overall, amputees were satisfied with their quality of life. The frequency of phantom pain seemed to be negatively associated with lower scores of the physical dimensions of participants’ quality of life. Researchers and practitioners should consider the clinical aspects and management of phantom pain due to its significant impact on patients’ health. Moreover, future studies should employ different recruitment strategies to reach out to amputees, especially in medium- and small-sized areas and regions.
